# Effect of Blood Nitrite and Nitrate Levels on Murine Platelet Function

**DOI:** 10.1371/journal.pone.0055699

**Published:** 2013-02-01

**Authors:** Ji Won Park, Barbora Piknova, Paul L. Huang, Constance T. Noguchi, Alan N. Schechter

**Affiliations:** 1 Molecular Medicine Branch, National Institute of Diabetes and Digestive and Kidney Diseases, National Institutes of Health, Bethesda, Maryland, United States of America; 2 Massachusetts General Hospital, Boston, Massachusetts, United States of America; University Heart Center Freiburg, Germany

## Abstract

Nitric oxide (NO) appears to play an important role in the regulation of thrombosis and hemostasis by inhibiting platelet function. The discovery of NO generation by reduction of nitrite (NO_2_^−^) and nitrate (NO_3_^−^) in mammals has led to increased attention to these anions with respect to potential beneficial effects in cardiovascular diseases. We have previously shown that nitrite anions at 0.1 µM inhibit aggregation and activation of human platelet preparations *in vitro* in the presence of red blood cells and this effect was enhanced by deoxygenation, an effect likely due to NO generation. In the present study, we hypothesized that nitrite and nitrate derived from the diet could also alter platelet function upon their conversion to NO *in vivo*. To manipulate the levels of nitrite and nitrate in mouse blood, we used antibiotics, NOS inhibitors, low nitrite/nitrate (NOx) diets, endothelial NOS knock-out mice and also supplementation with high levels of nitrite or nitrate in the drinking water. We found that all of these perturbations affected nitrite and nitrate levels but that the lowest whole blood values were obtained by dietary restriction. Platelet aggregation and ATP release were measured in whole blood and the results show an inverse correlation between nitrite/nitrate levels and platelet activity in aggregation and ATP release. Furthermore, we demonstrated that nitrite-supplemented group has a prolonged bleeding time compared with control or low NOx diet group. These results show that diet restriction contributes greatly to blood nitrite and nitrate levels and that platelet reactivity can be significantly affected by these manipulations. Our study suggests that endogenous levels of nitrite and nitrate may be used as a biomarker for predicting platelet function and that dietary manipulation may affect thrombotic processes.

## Introduction

Platelets are discoid non-nucleated cells that circulate freely in the plasma and are highly reactive entities that play a critical role in hemostasis and thrombosis both as a surface for clotting of plasma proteins and as a component of the clot itself. A number of stimuli are known to stimulate these platelet activities by modulating expression of surface glycoproteins, shape change, granule secretion, adhesion, and aggregation [1]. For example, collagen, one of the major components in the vessel wall, is exposed at the site of injury and initiates platelet adhesion by interacting with platelets through their glycoprotein and integrin receptors [2]. Therefore, to maintain normal vascular flow and responses to injury, there must be a tight balance between pro- and anti-thrombotic signals within the circulation and platelet function is delicately regulated under physiological conditions.

NO is one of the potent inhibitors of platelet function. NO is generated from the amino acid L-arginine and molecular oxygen in the endothelium by endothelial NOS (eNOS) and can diffuse into the lumen of the vessel and enter blood cells [3], as well as diffuse to the smooth muscle of the arteries and arteriolar vessels. The ability of NO to regulate cGMP via activation of sGC and Ca^2+^ mobilization is thought be the main mechanism by which NO negatively affects platelet activity [4]. Other isoforms of NOS, including inducible NOS (iNOS) and neuronal NOS (nNOS) may also contribute to NO levels in more specialized ways [5], [6], [7]. In addition to NOS-derived NO production, an alternative pathway for NO generation was recently established. Inorganic anions, nitrate (NO_3_^−^) and nitrite (NO_2_^−^), once considered inert end products from NO generation, can be reduced to NO by non-enzymatic as well as enzymatic pathways [8], [9], [10], [11], [12], [13]. While the effect of endothelium-derived NO on platelet function is well examined by numerous studies [3], [14], [15], [16], little is known about the biological effects of NO from exogenous sources such as diet rich in nitrite and nitrate. It has been shown that nitrate absorbed from diet can be reduced to nitrite mainly by bacterial nitrate reductases in the oral cavity [17] and nitrite can be further reduced to NO in blood and tissues by several pathways including deoxyhemoglobin [11], [18], deoxymyoglobin [19], [20], xanthine oxidase [21], [22], [23], and non-enzymatic reduction in the presence of protons [24], [25] or vitamin C [26]. These reductive pathways are known to be greatly enhanced under hypoxic conditions while oxygen-dependent endogenous NOS does not work efficiently under hypoxic situations. Animal studies demonstrated the therapeutic effects of inorganic nitrite and nitrate in ischemic injury models [27], suggesting that nitrite and nitrate have bioactivity that can partly compensate for the decrease of NOS-derived NO production under hypoxic conditions. In addition, we recently demonstrated that nitrite ions at 0.1 µM could inhibit aggregation and activation of human platelets in the presence of erythrocytes through its reduction to NO and this effect was promoted by deoxygenation since deoxyhemoglobin efficiently reduces nitrite to NO [28]. This suggests that nitrite might play a critical role in regulating platelet reactivity especially in small vessels under hypoxic conditions.

Considering the potential effect of blood and tissue nitrite and nitrate levels after their conversion to NO, it is important to study how those anions play a role in platelet physiology in intact mammals to see if variations in these levels would affect platelet reactivity and perhaps the dynamics of the blood clotting mechanisms. In the current study, we analyzed platelet aggregation and ATP release from dense granules as a function of nitrite and nitrate levels to study the role of these NO metabolites in regulating platelet activity in mice. We observed an inverse correlation between platelet activity and NO metabolite levels in whole blood. These results imply that NO metabolites, nitrite and nitrate, have a negative impact on platelet function presumably through their reduction to NO and may contribute to the regulation of platelet reactivity in living animals.

## Materials and Methods

### Ethics statement

The animal study protocol (#K049-MMB-09) was reviewed and approved by the NIDDK Animal Care and Use Committee.

### Materials

Bacitracin was purchased from X-GEN Pharmaceuticals, Inc. (Big Flats, NY), neomycin was purchased from Med-Pharmex (Carrollton, TX), tetracycline was purchased from Teva Pharmaceuticals (North Wales, PA). Collagen, ADP, ATP and luciferin-luciferase reagent (Chrono-Lume) were purchased from Chrono-log Corporation (Havertown, PA). All other reagents are purchased from Sigma (St Louis, MO).

### Animals and whole blood collection

C57BL/6 mice (10–12 weeks of age) were used as wild type (Jackson Laboratory, Bar Harbor, ME) compared with age-matched eNOS knock-out mice (eNOS^−^/^−^; provided by Dr. Paul Huang). Animals were maintained on a standard rodent chow diet (NIH07) with tap water. Whole blood was obtained by cardiac puncture and mixed with citrate-dextrose solution (Sigma, St. Louis, MO) with a 10∶1 ratio.

### Dietary control of nitrite and nitrate

For dietary depletion of nitrite and nitrate, the standard diet and tap water were switched to a low nitrite/nitrate (low NOx) diet (TD99366, Harlan Teklad, South Easton, MA) and MilliQ water for a week. For dietary supplementation study, sodium nitrite and sodium nitrate (Sigma, St. Louis, MO) were added to the drinking water of mice for a week at 0.1 g/L and 1 g/L concentration, respectively and mice were kept on the standard diet. To inhibit NOS activity, L-NAME (1 g/L) was added in the drinking water. For inhibition of bacterial nitrite/nitrate reductases, a combination of bacitracin (4 mg/ml), neomycin (4 mg/ml) and tetracycline (1 mg/ml) was used in the drinking water as a regimen of broad spectrum antibiotics to reduce mouse gut flora.

### Determination of whole blood nitrite and nitrate

Nitrite and nitrate levels in mouse whole blood were measured using a standard chemiluminescence method. For nitrite analysis, whole blood was mixed with nitrite preserving solution (K_3_Fe(CN)_6_, N-ethylmaleimide, water, Nonidet P-40) to maintain nitrite levels and kept frozen at −70°C until analysis. Samples were deproteinized by dilution with the same amount of methanol, then centrifuged for 5 min at 13,000 rpm (AccuSpinMicroR, Fisher Scientific, Pittsburgh, PA) and the supernatant was immediately injected into the chemiluminescent nitric oxide analyzer (NOA, Sievers, Model 280 NO analyzer, Boulder, CO) using helium as the carrier gas for chemiluminescence determination of NO. For nitrate analysis, whole blood in nitrite preserving solution was further diluted with deionized water at a 1∶9 ratio between the blood and water. At the time of sample analysis, a 3∶1 dilution of ethanol and thawed sample was centrifuged, and the supernatant was analyzed with NOA using the vanadium(III) chloride (VCl_3_) and chemiluminescence assay.

### Whole blood platelet aggregometry and ATP release analysis

Aggregation was analyzed by monitoring the changes of electrical impedance in ohm (Ω) to measure maximal aggregation response and by calculating the area under curve (AUC) to determine the total response duration using an aggregometer (Model 700, Chrono-Log Corporation, Havertown, PA). AUC represents the area under the 5-min aggregation curve and was generated by AGGRO/LINK8 program. The ATP released from platelets was monitored using a luciferin-luciferase assay. Chronolume® (Chrono-Log Corporation, Havertown, PA), a luciferin-luciferase reagent that contains firefly extract luciferase, was preincubated with diluted whole blood for 2 min at 37°C. The amount of released ATP was automatically calculated from the luminescent signal of an ATP standard. Platelet activation was induced by various concentrations of collagen or ADP and recorded for 5 min at 37°C.

### Measurement of bleeding time

C57BL/6 mice were anesthetized with isoflurane and body temperature of mice was maintained at 37°C by placing the mice on heating pads. The tail was amputated 0.5 cm from the tip. Blood was blotted onto filter paper every 10 seconds.

### Statistical Analysis

Statistics were analyzed using ANOVA (p-value<0.05, statistically significant) with Origin 8 (Origin Lab Corporation, Northampton, MA).

## Results

**Figure 1:**
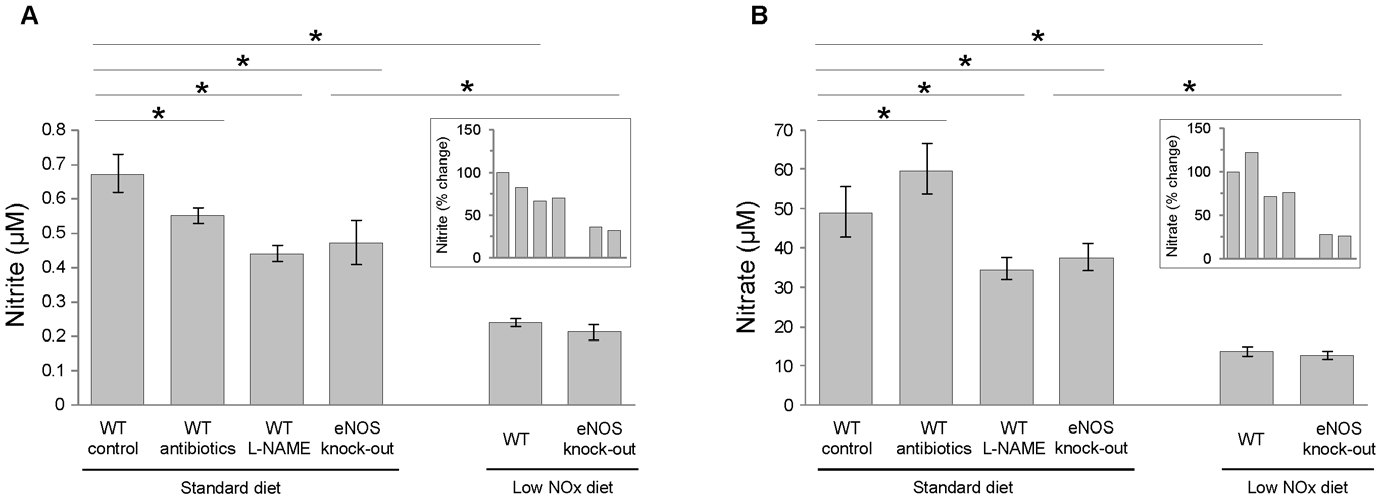
Influences of different perturbations in NO metabolism on nitrite and nitrate levels in whole blood. C57BL/6 mice were randomly divided into four groups and treated with antibiotics (bacitracin (4 mg/ml), neomycin (4 mg/ml), tetracycline (1 mg/ml)) or L-NAME (1 g/L) or low nitrite/nitrate (NOx) diet for a week. Age-matched eNOS knock-out mice were used. Nitrite (A) and nitrate (B) were measured using a chemiluminescence NO analyzer (Sievers, Model 280 NO analyzer). Relative values to wild-type control on standard diets were calculated in each treatment group and inserted in both (A) and (B). Data are means ± SEM (n≥7). *p<0.05.

NO metabolism in the body can be attributed to two different mechanisms, namely endogenous NOS-dependent pathways and exogenous diet-dependent pathways. To analyze the contribution of several factors that affect NO metabolic system to the levels of nitrite and nitrate in whole blood, we employed a mouse system in which we can manipulate the levels of NO metabolites. In Fig. 1, we analyzed nitrite and nitrate levels in whole blood of groups of mice with different dietary treatments. Our animals have whole blood levels of nitrite at 0.67±0.05 µM and of nitrate at 49±6 µM under control conditions. Inhibition of bacterial nitrite/nitrate reductases by antibiotic treatment caused a decrease in nitrite but increase in nitrate, 18% decrease and 22% increase, respectively. However, inhibition of endogenous NOS by L-NAME or genetic disruption of eNOS showed more significant changes in nitrite levels as well as in nitrate levels (∼30% decrease). The most prominent changes in nitrite and nitrate levels were obtained by the restriction of the diet. Low NOx diet treatment for a week resulted in dramatic decreases in both nitrite (64%) and nitrate (72%) in WT mice and there was no clear difference between WT and eNOS knock-out in nitrite and nitrate levels under low NOx diet.

**Figure 2:**
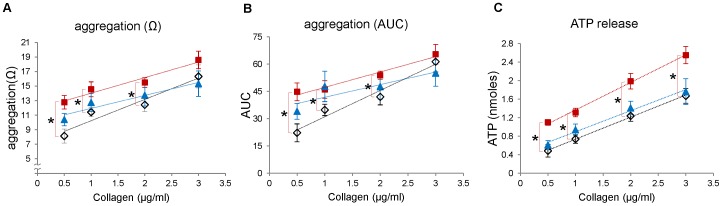
Whole blood platelet aggregometry and ATP release in response to collagen. C57BL/6 mice were given either standard diet or low NOx diet for a week and age-matched eNOS knock-out mice were also given standard diet for a week. Whole blood was diluted with saline (1∶4) and preincubated with Chronolume® for 2 min at 37°C for ATP measurement. The aggregation was induced by collagen (0.5∼3 µg/ml) addition and expressed in maximal impedance (A) or area under the 5-min aggregation curve (AUC, B). The ATP released from platelets was measured with a luciferin-luciferase assay and the amount of ATP was calculated using an ATP standard (C). Wild-type on standard diets: open diamond, wild-type on low NOx diets: red square, eNOS knock-out on standard diets: blue triangle. Data are means ± SEM (n≥5). *p<0.05 compared with wild-type on standard diets at each concentration of collagen.

**Figure 3:**
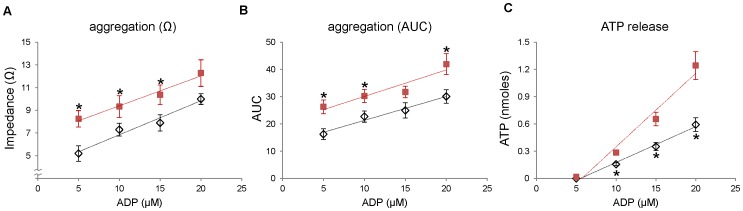
Whole blood platelet aggregometry and ATP release in response to ADP. C57BL/6 mice were divided into two groups and one group was given standard diets and the other group was given low NOx diets for a week. Whole blood was taken by cardiac puncture and diluted with saline (1∶4). Chronolume® was preincubated with diluted blood for 2 min at 37°C for ATP measurement and then aggregation was induced by ADP (5∼20 µM) addition. Aggregation amplitude was shown as maximal impedance (A) and the total response duration was expressed as area under the 5-min aggregation curve (AUC, B). ATP release was analyzed by luciferin-luciferase assay using an ATP standard (C). Standard diets: open diamond, low NOx diets: red square. Data are means ± SEM (n≥6). * p<0.05 compared with standard diet group at each concentration of ADP.

Next, to examine whether the nitrite and nitrate levels in blood are correlated with the function of platelets, we measured platelet aggregation and ATP secretion upon stimulation with soluble collagen in three different mouse groups; wild type C57BL/6 mouse on a standard diet (control group), eNOS-deficient mouse on a standard diet (eNOS knock-out group), and wild type C57BL/6 mouse on a low nitrite/nitrate (NOx) diet (low NOx group). The result from eNOS knock-out group represents the contribution of endogenous eNOS pathway in NO production and subsequent metabolism of NO to nitrite and nitrate, whereas the low NOx group presumably represents the contribution of diet-derived NO and its metabolite production through the serial reduction of nitrate. Platelet aggregation and ATP secretion were measured simultaneously in response to collagen stimulation (Fig. 2). At low concentrations of collagen (0.5∼1 µg/mL), both aggregation and ATP release were slightly enhanced in eNOS-deficient mouse blood although the changes did not reach statistical significance, but there was no difference in aggregation and ATP release at higher (2∼3 µg/mL) concentrations of collagen. However, we observed more significant enhancement in aggregation and ATP release in the low NOx diet group at all concentrations of collagen used (0.5∼3 µg/mL) compared with control group. We confirmed this enhanced platelet reactivity in aggregation and ATP release of the low NOx group in response to broad concentrations of ADP stimulation (Fig. 3).

**Figure 4:**
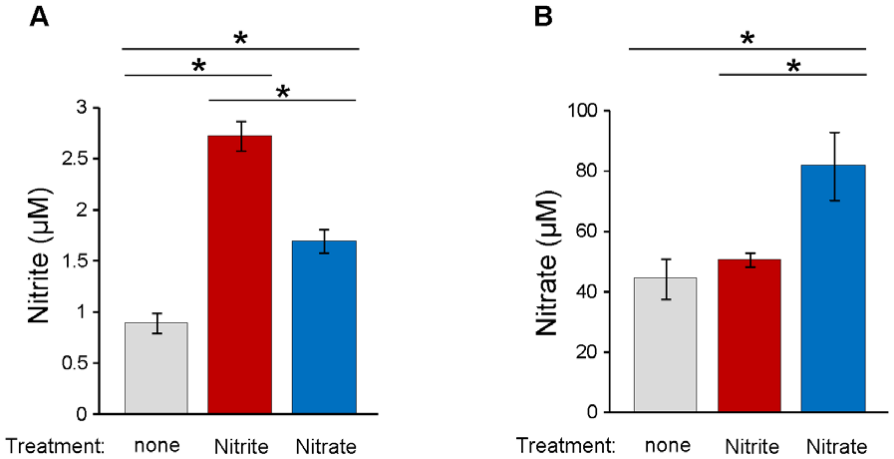
Effect of nitrite and nitrate supplementation on the levels of nitrite and nitrate in whole blood. C57BL/6 mice were divided into three groups. Control group received normal water (no treatment), second group received nitrite (0.1 g/L)-containing water and the third group received nitrate (1 g/L)-containing water for a week. All groups were fed standard diets during the treatment period. Nitrite (A) and nitrate (B) in the whole blood from each group were measured using a chemiluminescence NO analyzer (Sievers, Model 280 NO analyzer). Data are means ± SEM (n≥4). * p<0.05.

**Figure 5:**
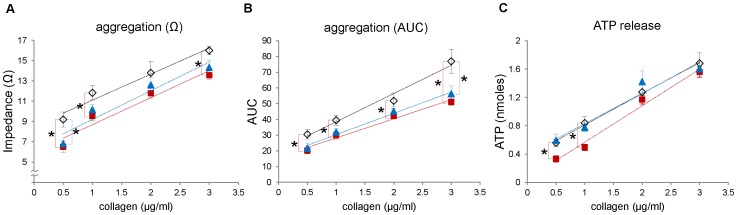
Effect of nitrite and nitrate supplementation on platelet reactivity in whole blood. Whole blood from the control (no treatment) mice or the mice that were given additional nitrite (0.1 g/L) or nitrate (1 g/L) in the drinking water for a week was taken and diluted with saline (1∶4) for aggregation (A and B) and ATP release (C) measurement. Chronolume® was added to the diluted blood for 2 min at 37°C for ATP measurement and then platelets were activated by collagen (0.5∼3 µg/ml) addition. Aggregation amplitude was shown as maximal impedance (A) and the total response duration was expressed as area under the 5-min aggregation curve (AUC, B). The amount of ATP released from platelets was analyzed by luciferin-luciferase assay using an ATP standard (C). Control (no treatment): open diamond, nitrite (0.1 g/L): red square, nitrate (1 g/L): blue triangle. Data are means ± SEM (n≥5). * p<0.05 compared with control (no treatment) at each concentration of collagen.

To further study the effect of dietary source of nitrite and nitrate on aggregation, we supplied nitrite (0.1 g/L) or nitrate (1 g/L)-containing water to the mice for a week and then measured aggregation and ATP secretion as well as nitrite/nitrate levels in blood (Fig. 4 and 5). The levels of nitrite in whole blood after the treatments increased greatly in both nitrite and nitrate treatment groups indicating that the administered nitrate was reduced to nitrite (Fig. 4A). Nitrate levels in the nitrate treatment group showed a significant increase as expected while nitrite treatment did not show an increase in nitrate levels (Fig. 4B). Although high levels of nitrite were administered to this group, it was not reflected in nitrate levels because endogenous nitrate levels were already forty-fold higher than endogenous nitrite levels before nitrite treatment. Platelet aggregation and ATP release were measured in whole blood of nitrite- or nitrate-treated mice (Fig. 5A∼C). In contrast with the results from the low NOx diet group, both nitrite and nitrate treatment inhibited collagen-induced aggregation and ATP release was also diminished in nitrite-treated mice.

**Figure 6:**
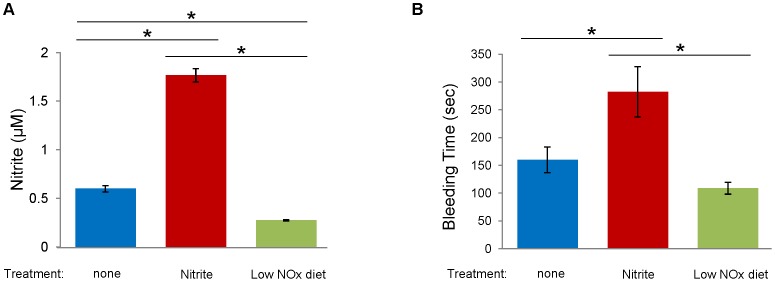
Measurement of tail bleeding time. Mice were divided into three groups for 1-week treatment; control (no treatment, standard diets), nitrite supplementation (0.1 g/L) and low NOx diet group. Nitrite in the whole blood from each group of mice was measured using a chemiluminescence NO analyzer (A) (Sievers, Model 280 NO analyzer). The tail was amputated 0.5 cm from the tip and blood was blotted onto filter paper every 10 seconds (B). Data are means ± SEM (n≥8). *p<0.05.

In order to examine the physiological consequences of blood nitrite levels in regulating platelet function, we measured tail bleeding time in two different treatment groups in which additional nitrite was administered or nitrite/nitrate ingestion was restricted, compared with control (no treatment) group (Fig. 6B). Nitrite-supplemented group showed the longest bleeding time, in contrast, low NOx diet group exhibited the shortest bleeding time although the bleeding time of low NOx diet group was not statistically different from that of control group (p = 0.075). Nitrite levels in whole blood from these groups were analyzed (Fig. 6A) and we confirmed that nitrite supplementation increased nitrite levels from 0.60±0.03 to 1.77±0.07 µM and low NOx diet treatment decreased nitrite levels to 0.28±0.01 µM.

## Discussion

The roles of nitrite and nitrate as exogenous NO sources have recently obtained increasing attention with respect to their potential beneficial effects in cardiovascular diseases [29], [30], [31]. Although the endogenous NOS system partially contributes to the changes of nitrite and nitrate levels, a few animal studies [32], [33], [34], in which low NOx diet was used for dietary nitrite and nitrate depletion, show that blood and tissue nitrite and nitrate levels were significantly reduced under the low NOx diet suggesting the remarkable contribution of diet to steady-state levels of nitrite and nitrate.

Since the locally available amount of vascular NO is critical to delicate regulation of platelet function, we hypothesized that exogenous nitrite and nitrate could also have an impact on platelet reactivity once they are converted to NO. The fact that the endogenous NOS pathway becomes inactive but nitrate/nitrite reduction pathway is enhanced under certain situations such as hypoxia led us to pay more attention to the new paradigm for NO production which involves stepwise reduction of nitrate to NO. A human diet rich in vegetables (app. 300 g) is known to provide more nitrate than what is produced endogenously over a day by all three NOS enzymes [17], [35]. In agreement with this, our current result clearly shows that low NOx diet treatment decreased the levels of nitrite and nitrate in mouse whole blood more significantly as compared to NOS inhibition by L-NAME treatment in the drinking water (nitrite decrease; 64% vs. 34%, nitrate decrease; 72% vs. 29%, Fig. 1). It is interesting to note that the genetic deletion of eNOS showed similar effects to L-NAME treatment, which inhibits all three NOS isoforms, on the levels of both nitrite and nitrate in whole blood. Thus eNOS seems to be the major contributor to NO metabolism in the circulation rather than other isoforms of NOS in mice (Fig. 1), although it is not certain that L-NAME treatment inhibited the activity of all three NOS isoforms completely when administered in the drinking water. It is also notable that antibiotic treatment to inhibit bacterial enzymes that are responsible for nitrate/nitrite reduction affected whole blood nitrite and nitrate levels in the mice. Nitrate levels increased (22%) upon antibiotic treatment due to accumulation of nitrate obtained from the diet and oxidation of endogenously produced NO while nitrite levels decreased (18%) because nitrate reduction to nitrite was inhibited by antibiotics. In addition to commensal bacteria, the presence of functional mammalian nitrate reductases has been reported [36] and that study showed that the inhibition of xanthine oxidoreductase attenuated nitrate reducing activity in rodent and human tissues. Furthermore, it was reported that even germ free mice showed some degree of nitrate reducing activity and xanthine oxidoreductase expression level was upregulated in the liver homogenate of germ free mice [37]. However, when we provided eNOS knock-out mice with oxypurinol, a xanthine oxidoreductase inhibitor, in drinking water to see if xanthine oxidoreductase could affect NO metabolism by regulating nitrate/nitrite reduction pathways in eNOS knock-out mice, nitrite levels in whole blood, liver, kidney and brain did not change significantly (data not shown). Although the data on wild-type mice are not available at present, this result suggests xanthine oxidoreductase is not the main mechanism that regulates nitrite/nitrate homeostasis in eNOS knock-out mice.

It should also be noted that the basal levels of whole blood nitrite and nitrate in the C57BL/6 mice were significantly higher than levels we have found in human blood (nitrite 50∼300 nM, nitrate 20∼40 µM) [13]. It is necessary to be aware of the variations in these parameters among different species.

The major new observation in our current study is the clear differences between groups which were manipulated to have different nitrite/nitrate levels in *in vitro* platelet functional parameters (eg. platelet aggregation and granule secretion) and bleeding time in intact animals. These results suggest that the effects of nitrite we observed in *in vitro* studies of human platelets appear to occur *in vivo* in mice which may be used as an animal model to study the mechanisms involved. Although previous studies showed that endothelium-derived NO plays a critical role in inhibiting platelet function by stimulating cGMP production and subsequent activation of protein kinase G (PKG) which phosphorylates numerous proteins related to platelet function [38], little has been known about the biological effect of nitrite and nitrate derived from the diet on platelet reactivity. We have shown in this study that there is an inverse correlation between nitrite/nitrate levels and platelet function measured by whole blood platelet aggregometry, ATP release and bleeding time analysis. An earlier report by Richardson *et al.* suggested inorganic nitrate ingestion inhibited human platelet aggregation [39] and a more recent report by Webb *et al.* showed oral intake of nitrate-rich beetroot juice decreased platelet aggregation suggesting exogenous NO sources could have an inhibitory effect on human platelet function [40] but this was not interpreted as to its metabolism. Our study extended this notion by showing that manipulation of nitrite and nitrate levels in blood via diet restriction in the mice greatly affect platelet function since mice exhibit better platelet aggregation and ATP secretion under low NOx diet but showed diminished aggregation when additional nitrite or nitrate was provided. In contrast with low NOx diet group, eNOS knock-out mice did not show prominent differences in platelet aggregation and ATP release (Fig. 2). This is in accordance with a previous report by Tymvios *et al.* that the platelet aggregation *in vivo* to thrombin was not significantly different in eNOS knock-out mice compared with control mice [41]. Although Freedman *et al.* showed that the bleeding time of eNOS knock-out mice was decreased compared with that of control mice, they observed no significant difference in levels of surface expressed P-selectin upon stimulation in wild-type and eNOS knock-out mice [42] suggesting eNOS-derived NO plays a role after platelet activation by inhibiting platelet recruitment to the growing thrombus. Moreover, we demonstrated in this study that nitrite-supplemented group has a prolonged bleeding time compared with control or low NOx diet group. Together with the reduced aggregation results from nitrite-supplemented group, these results indicate that the levels of blood nitrite which can be controlled by our diet could substantially affect platelet reactivity and further contribute to regulation of hemostasis.

In conclusion, diet restriction of the amount of nitrite and nitrate affected the levels of nitrite and nitrate in whole blood to a great extent as compared to inhibition of NO generating enzymes in the body. In parallel with this, we found that nitrite and nitrate derived from the diet have a significant effect on protracting bleeding time as well as an inhibitory effect on platelet responses while genetic disruption of eNOS did not show statistically significant inhibition in platelet responses. These results imply that dietary nitrite and nitrate could be used to modulate hemostasis and thrombosis by regulating platelet reactivity.
